# Supplementary material for: Knowledge of Self-Isolation Rules in the UK for Those Who Have Symptoms of COVID-19: A Repeated Cross-Sectional Survey Study
Source: Int J Environ Res Public Health. 2023 Jan 20;20(3):1952. doi: 10.3390/ijerph20031952 (PMC9914733; doi:10.3390/ijerph20031952)
Supplement: Supplementary file 1 [file ijerph-20-01952-s001.zip › ijerph-1974416-supplementary.pdf]

Supplementary materials – measure of knowledge of Government rules if symptomatic

The Government has issued advice on how people should help prevent the spread of coronavirus if they have symptoms. If <u>you</u> have symptoms of coronavirus, you:

- can go out if your symptoms are mild (false)
- can go to the shops for groceries/pharmacy (false)*
- can go to the shops for things other than groceries/pharmacy (false)*
- can go for a walk or some other exercise (false)
- can go out to work if you cannot work from home (false)
- can go out for a medical need, or to donate blood (true)
- can go out to help or provide care for a vulnerable person (false)
- can go out if you're wearing a face covering (false)
- can go out to meet up with friends and/or family that you don't live with, <u>indoors</u> (false)*
- can go out to meet up with friends and/or family that you don't live with, <u>outdoors</u> (false)
- can go out to spend time outdoors for recreational purposes (including to sit in parks etc) (false)
- should self-isolate (true)
- could be eligible for self-isolation payments (if you are on benefits and/or a lower income and cannot work from home) (true)
- can go out to get a test to see if you have coronavirus (true)
- can have someone who you don't live with over to your home (false)*
- should get a test, but can go out as normal while you are waiting for the result (false) [included in waves 32 to 50]
- should take a rapid 'lateral flow' coronavirus test (results within 30 minutes) (false) [included in waves 51 to 52]
- should take a lab-processed 'PCR' coronavirus test (results typically within a day or two) (true) [included in waves 51 to 52]
- should take a test (true) [included in waves 53 to 68]

SCALE

- True
- False
- Don't know

* Included in derived knowledge measure that would involve contact with people from other households.

<u>Alternative phrasing of the question, used in waves 47 and 48 (split sample, half of participants)</u>

The Government has issued advice on how people should help prevent the spread of coronavirus if they have symptoms. Please tell us, for the following options, if you think they are true or false?

If someone develops symptoms of coronavirus, they:

- can go out if their symptoms are mild (false)
- can go to the shops for groceries/pharmacy (false)*
- can go to the shops for things other than groceries/pharmacy (false)*
- can go for a walk or some other exercise (false)
- can go out to work if they cannot work from home (false)
- can go out for a medical need, or to donate blood (true)
- can go out to help or provide care for a vulnerable person (false)
- can go out if they are wearing a face covering (false)
- can go out to meet up with friends and/or family they don't live with, indoors (false)*
- can go out to meet up with friends and/or family they don't live with, outdoors (false)
- can go out to spend time outdoors for recreational purposes (including to sit in parks etc) (false)
- should self-isolate (true)
- could be eligible for self-isolation payments (if they are on benefits and/or a lower income and cannot work from home) (true)
- can go out to get a test to see if they have coronavirus (true)
- can have someone who they don't live with over to their home (false)*
- should get a test, but can go out as normal while they are waiting for the result (false)

SCALE

- True
- False
- Don't know
